# Defining the Functional Interactome of Spliceosome-Associated G-Patch Protein Gpl1 in the Fission Yeast *Schizosaccharomyces pombe*

**DOI:** 10.3390/ijms232112800

**Published:** 2022-10-24

**Authors:** Tomas Selicky, Matus Jurcik, Barbora Mikolaskova, Alexandra Pitelova, Nina Mayerova, Miroslava Kretova, Michaela Osadska, Jan Jurcik, Roman Holic, Lenka Kohutova, Jana Bellova, Zsigmond Benko, Juraj Gregan, Silvia Bagelova Polakova, Peter Barath, Lubos Cipak, Ingrid Cipakova

**Affiliations:** 1 Department of Genetics, Cancer Research Institute, Biomedical Research Center, Slovak Academy of Sciences, Dubravska cesta 9, 845 05 Bratislava, Slovakia; 2 Department of Membrane Biochemistry, Institute of Animal Biochemistry and Genetics, Centre of Biosciences, Slovak Academy of Sciences, Dubravska cesta 9, 840 05 Bratislava, Slovakia; 3 Department of Genetics, Faculty of Natural Sciences, Comenius University in Bratislava, 841 04 Bratislava, Slovakia; 4 Department of Glycobiology, Institute of Chemistry, Slovak Academy of Sciences, Dubravska cesta 9, 845 38 Bratislava, Slovakia; 5 Department of Molecular Biotechnology and Microbiology, University of Debrecen, Egyetem tér 1, H4032 Debrecen, Hungary; 6 Department of Applied Genetics and Cell Biology, Institute of Microbial Genetics, University of Natural Resources and Life Sciences, Vienna (BOKU), Konrad Lorenz Strasse 24, 3430 Tulln an der Donau, Austria; 7 Medirex Group Academy, Novozamocka 67, 949 05 Nitra, Slovakia

**Keywords:** Gpl1, Gih35, Wdr83, tandem affinity purification, pre-mRNA splicing, *Schizosaccharomyces pombe*

## Abstract

Pre-mRNA splicing plays a fundamental role in securing protein diversity by generating multiple transcript isoforms from a single gene. Recently, it has been shown that specific G-patch domain-containing proteins are critical cofactors involved in the regulation of splicing processes. In this study, using the knock-out strategy, affinity purification and the yeast-two-hybrid assay, we demonstrated that the spliceosome-associated G-patch protein Gpl1 of the fission yeast *S. pombe* mediates interactions between putative RNA helicase Gih35 (SPAC20H4.09) and WD repeat protein Wdr83, and ensures their binding to the spliceosome. Furthermore, RT-qPCR analysis of the splicing efficiency of deletion mutants indicated that the absence of any of the components of the Gpl1-Gih35-Wdr83 complex leads to defective splicing of *fet5* and *pwi1*, the reference genes whose unspliced isoforms harboring premature stop codons are targeted for degradation by the nonsense-mediated decay (NMD) pathway. Together, our results shed more light on the functional interactome of G-patch protein Gpl1 and revealed that the Gpl1-Gih35-Wdr83 complex plays an important role in the regulation of pre-mRNA splicing in *S. pombe*.

## 1. Introduction

Many precursor messenger RNA molecules (pre-mRNAs) contain introns that need to be removed in order to obtain mature mRNAs that can serve as a template for translation [1]. The removal of introns from pre-mRNAs is catalyzed by the spliceosome. The spliceosome is one of the largest complexes in the cell, comprising hundreds of proteins and factors linked by uncounted interactions. Importantly, dozens of proteins and factors that act as splicing activators or repressors interact with the spliceosome only transiently at specific stages of its assembly and rearrangement, making the regulation of splicing processes particularly complex [2].

Among the many splicing factors, RNA helicases have been identified as important regulators of splicing processes. They are implicated in promoting conformational rearrangements and ensure that only appropriate substrates proceed through the splicing reactions. Their activities can be regulated by auto-inhibitory domains that maintain their low activities until binding to correct substrates [3,4], by additional domains that recognize specific RNA features, thus directing them to appropriate RNA substrates [5,6], by post-translation modifications [7,8,9], and through their interactions with other proteins and factors [10,11,12,13,14].

Recently, it has been shown that specific proteins, known as G-patch proteins, are critical cofactors of RNA helicases. These proteins contain a unique G-patch domain characterized by the presence of six highly conserved glycine residues. The domain is approximately 45–50 amino acids in length with the signature *hhx*(3)G*ax*(2)G*x*G*h*G*x*(4)G, where *h* stands for a hydrophobic residue, *a* is an aromatic residue and *x*(n) is a number of positions occupied by any nonconserved residues. According to secondary structure predictions, the G-patch domain is composed of two α-helices, with four out of the six conserved glycines located within an intervening loop [15].

So far, several G-patch proteins have been shown to interact with RNA helicases. For instance, in human cells, G-patch domain-containing proteins GPKOW, NKRF, PINX1, RBM5, RBM17, TFIP11 and ZGPAT have been linked with the regulation of activities of helicases DHX15 (human Prp43 ortholog) and DHX16 (human Prp2 ortholog) [16,17,18,19,20,21,22,23,24]. In *Saccharomyces cerevisiae*, G-patch proteins Cmg1, Ntr1, Pxr1 and Sqs1 were identified to bind to the multifunctional helicase Prp43 to regulate its ATPase and helicase activities [25,26,27,28,29,30,31,32,33]. Concerning the Ntr1 in the budding yeast *S. cerevisiae*, it was shown to stimulate the RNA unwinding activity of Prp43 and promote the release of excised introns from splicing complexes possibly assisted by Ntr2 [31,34,35]. Similarly, *S. cerevisiae* G-patch protein Spp2 was found to bind to the C-terminal part of helicase Prp2, thus regulating its RNA-dependent ATPase activity [36,37,38,39]. In the fission yeast *S. pombe*, at least eleven proteins were annotated to contain the G-patch domain (Cwf28, Gpl1, Ntr1, Pxr1, Rbm10, Rbm17, SPAC6F6.19, SPBC1604.16c, Sqs1, Sqs2 and Tma23) (ontology term: G-patch domain, PBO:0000605; https://www.pombase.org/term/PBO:0000605 (accessed on 15 August 2022)) [40]. Despite the importance of G-patch proteins in the regulation of RNA helicases, the functions and interactions of most of the *S. pombe* G-patch proteins remain uncharacterized.

Recently, we reported that the G-patch protein Gpl1 in the fission yeast *S. pombe* interacts with proteins involved in pre-mRNA splicing [41,42,43]. Previous studies also have shown that depletion of Gpl1 causes canonical splicing defects with broad increases in pre-mRNA species and decreases in mature mRNA species [44]. These findings characterized Gpl1 as a novel protein involved in the regulation of pre-mRNA splicing in *S. pombe*.

In this study, we provided new insights into protein–protein interactions between G-patch protein Gpl1, putative RNA helicase Gih35 (Gih35 stands for *G*pl1 *i*nteracting *h*elicase Dhx*35*) and WD repeat protein Wdr83, the spliceosome-associated factors, which, based on our findings, form a tripartite Gpl1-Gih35-Wdr83 complex. Gih35 helicase belongs to the family of DEAD/DEAH-box helicases. These helicases are highly conserved RNA-binding proteins with ATPase activity and are crucial for RNA metabolism [45]. Recently, the human ortholog of Gih35, known as DHX35, has been identified as part of spliceosomal complex C [46]. Similarly, the human ortholog of WD repeat protein Wdr83, which is known as MORG1 or WDR83, was also shown to be part of spliceosomal complex C [47].

As Gpl1, Gih35 and Wdr83 are non-essential for cell viability, we were able to confirm the critical role of Gpl1 in the formation and stability of the Gpl1-Gih35-Wdr83 complex, and also for its binding to the spliceosome. Importantly, we showed that deletion of any of the Gpl1-Gih35-Wdr83 complex components leads to defective splicing of pre-mRNA. Based on these findings, we proposed that Gpl1 secures the stability of the Gpl1-Gih35-Wdr83 complex and allows its binding to the spliceosome, thus regulating the splicing processes in *S. pombe*.

## 2. Results

### 2.1. Gpl1, Gih35 and Wdr83 Form a Complex That Associates with the Spliceosome

**Figure 1:**
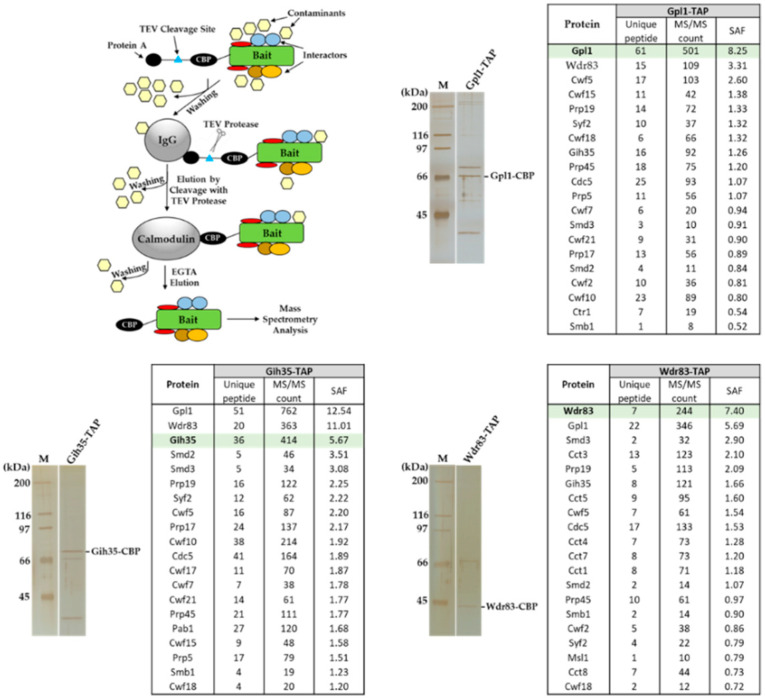
Identification of proteins co-purifying with Gpl1-TAP, Gih35-TAP and Wdr83-TAP proteins. Proteins associated with Gpl1, Gih35 and Wdr83 were isolated from cycling *S. pombe* cells by tandem affinity purification, separated by SDS-PAGE, visualized by silver staining and analyzed by mass spectrometry. Selected TOP20 interactors sorted according to their SAF (spectral abundance factor) are presented here. For a full list of co-purified proteins, see Table S1.

Previously, we showed that *S. pombe* Gpl1, which was found to be required for efficient splicing [44], co-purifies with a putative ATP-dependent RNA helicase Gih35, WD repeat protein Wdr83 and several other splicing factors [42]. To characterize the functional interactome of Gpl1, we performed affinity purifications of Gpl1, Gih35 and Wdr83 and identified co-purifying proteins. We found that all three proteins, namely, Gpl1, Gih35 and Wdr83, are present among the top co-purified interactors (Figure 1). Additionally, proteins of Prp19 and U5 snRNP complexes, such as ubiquitin-protein ligase E4 Prp19 and Prp19 complex subunit Cdc5, and U5 snRNP complex subunit Spp42 and U5 snRNP GTPase subunit Cwf10, as well as some other splicing machinery proteins, were found to co-purify within the isolated complexes. Interestingly, U5 snRNP complex ATPase subunit Brr2 and ATP-dependent RNA helicases Mtl1, Prp22 and Prp43 were also found to be part of isolated complexes (Table S1). These findings raise the possibility that non-essential proteins Gpl1, Gih35 and Wdr83 associate with the spliceosome as part of the Gpl1-Gih35-Wdr83 complex.

### 2.2. Gpl1 Mediates Interactions between Gih35 and Wdr83

**Figure 2:**
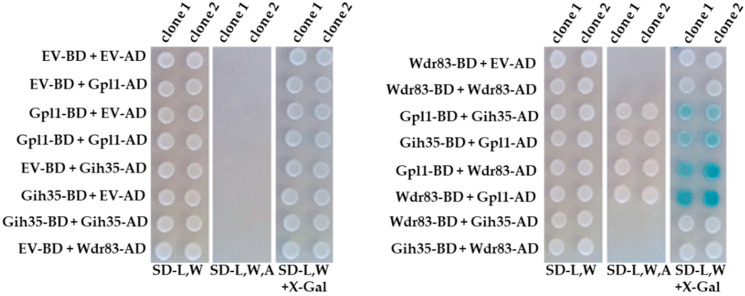
Physical protein–protein interactions between Gpl1, Gih35 and Wdr83 proteins analyzed by the Y2H assay. EV-BD or EV-AD and protein-BD or protein-AD indicate empty Y2H vector bearing binding or activation domains and Y2H vectors expressing protein of interests fused with binding or activation domains, respectively. SD-L,W—synthetic dropout media without leucine and tryptophan; SD-L,W,A—synthetic dropout media without leucine, tryptophan and adenine; SD-L,W—synthetic dropout media without leucine and tryptophan and supplemented with X-gal.

To validate the above findings and to analyze the protein–protein interactions between the components of the Gpl1-Gih35-Wdr83 complex in detail, we performed the yeast-two-hybrid assay (Y2H) (Figure 2). Y2H analyses revealed that Gpl1 interacts with RNA helicase Gih35 and WD repeat protein Wdr83. However, no interaction between Gih35 and Wdr83 was detected. These findings raised an interesting possibility that Gpl1 may function as a bridging protein that holds together Gih35 and Wdr83 within the Gpl1-Gih35-Wdr83 complex.

### 2.3. Domain-Specific Interactions of Gpl1 with Gih35 and Wdr83

Intriguingly, the ability of Gpl1 to function as a protein that allows Gih35 and Wdr83 to be part of the Gpl1-Gih35-Wdr83 complex, prompted us to map its interaction domains. For this purpose, we prepared a panel of Gpl1 truncations and tested them for binding to Gih35 and Wdr83.

**Figure 3:**
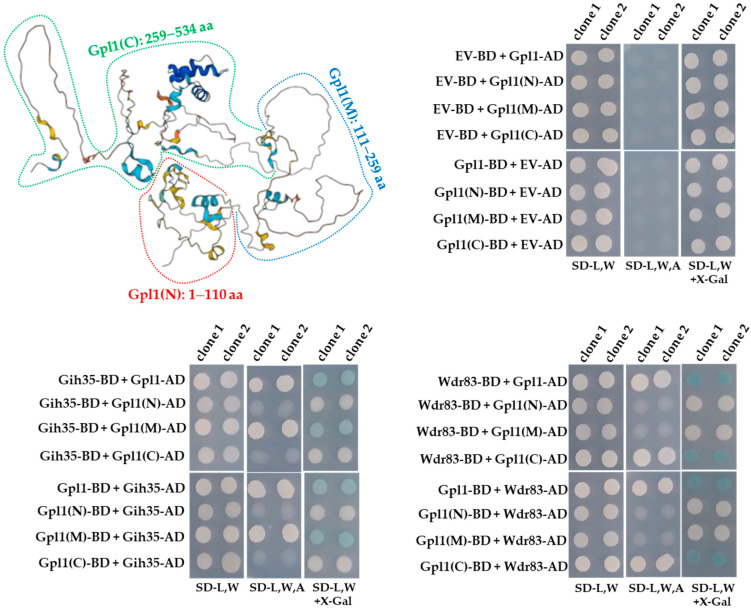
Predicted structure of Gpl1 and Y2H analysis of interactions between Gpl1 and its truncated forms and Gih35 or Wdr83 proteins. The three-dimensional structure of Gpl1 was predicted using AlphaFold [48,49] and downloaded from the AlphaFold Protein Structure Database (https://alphafold.com/entry/Q9HE07, accessed on 28 July 2022). The truncated forms are indicated (Gpl1(N): 1–110 aa, red dashed line; Gpl1(M): 111–259 aa, blue dashed line; Gpl1(C): 259–534 aa, green dashed line). G-patch domain is located within the N-terminal region of Gpl1 (36–110 aa). EV-BD or EV-AD and protein-BD or protein-AD indicate empty Y2H vector bearing binding or activation domains and Y2H vectors expressing protein of interest fused with binding or activation domains, respectively. SD-L,W—synthetic dropout media without leucine and tryptophan; SD-L,W,A—synthetic dropout media without leucine, tryptophan and adenine; SD-L,W—synthetic dropout media without leucine and tryptophan and supplemented with X-gal.

The Y2H constructs were designed to express the N-terminal region (Gpl1(N), 1–110 aa), the middle region (Gpl1(M), 111–259 aa) and the C-terminal region (Gpl1(C), 259–534 aa) of the Gpl1 protein (Figure 3). We found that Gih35 binds to the full-length Gpl1 protein and to its middle region (111–259 aa). On the other side, we detected interactions between Wdr83 and full-length Gpl1 as well as its C-terminal region (259–534 aa). This revealed that Gih35 and Wdr83 bind to distinct parts of Gpl1.

### 2.4. Gpl1 Is Important for Binding the Gpl1-Gih35-Wdr83 Complex to the Spliceosome

**Figure 4:**
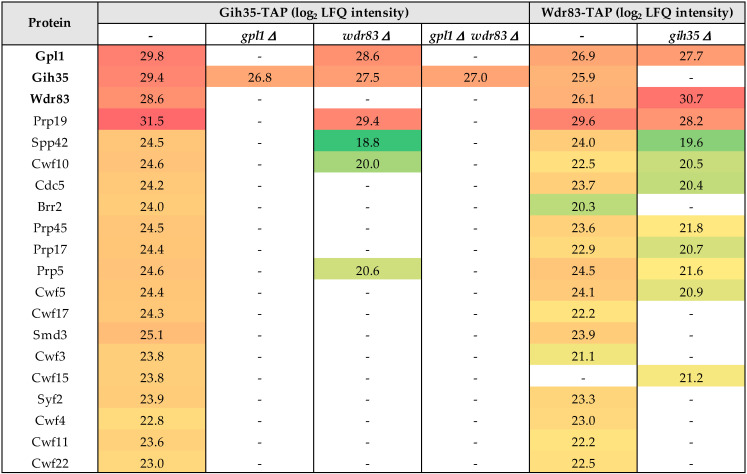
List of selected proteins with determined label-free quantification (LFQ) intensities (see Material and Methods) co-purified with Gih35-TAP and Wdr83-TAP from wild-type cells and indicated deletion mutants. For a full list of identified proteins and their post-translational modifications, see Table S2.

Next, we wanted to find out how the Gpl1-Gih35-Wdr83 complex binds to the spliceosome. Therefore, we performed a series of tandem affinity isolations of Gpl1, Gih35 and Wdr83 complexes from cells bearing a particular single *gpl1*, *gih35* or *wdr83* deletions or double *gpl1* and *wdr83* deletion. We found that single deletions or double deletions of a particular component of the Gpl1-Gih35-Wdr83 complex led to significant changes in the interactomes of isolated proteins (Figure 4; Table S2). Specifically, the Gih35 interactome was compromised for Wdr83 and for most of the splicing factors when isolated from cells deleted for *gpl1*. Similarly, most of the splicing factors diminished from the Gih35 interactome when both *gpl1* and *wdr83* were deleted. Interestingly, RNA helicase Gih35 still co-purified with Gpl1 and some of the splicing factors when *wdr83* was deleted. The observed changes in the interactomes of Gpl1-Gih35-Wdr83 complex deletion mutants were further supported by analysis of the Wdr83 complex isolated from cells deleted for *gih35*. In this case, the protein Gpl1, as well as most of the proteins of the Prp19 complex, co-purified with Wdr83. These results indicated that the Gih35 helicase is part of the Gpl1-Gih35-Wdr83 complex, but to associate with the spliceosome, it requires the interaction with Gpl1. Altogether, these findings confirmed the above results of the Y2H assay and provided further support for the hypothesis that Gpl1 functions as a bridging protein for Gih35 and Wdr83 on one side, and as an anchoring protein that allows the binding of the Gpl1-Gih35-Wdr83 complex to the spliceosome on the other side.

### 2.5. Expression of Proteins Forming the Gpl1-Gih35-Wdr83 Complex Is Mutually Independent

**Figure 5:**
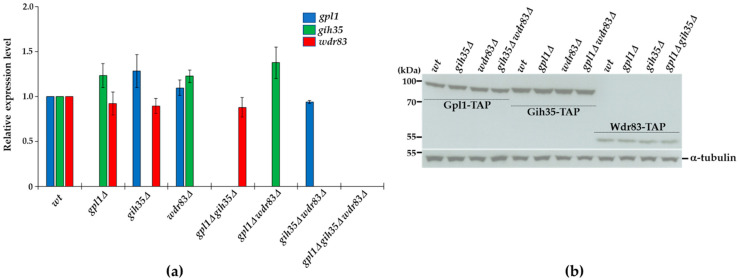
Analyses of gene expression and protein levels of components of the Gpl1-Gih35-Wdr83 complex: (**a**) RNA was isolated from wild-type (*wt*) cells or indicated deletion mutants in the exponential phase (OD_595_ = 0.5–0.6), and gene expression was analyzed using RT-qPCR. The data represent transcript levels relative to wild-type cells after normalization to actin. The plotted values are the mean of three independent biological replicates ± SEM (**b**) Wild-type cells or deletion mutants expressing Gpl1-TAP (85.19 kDa), Gih35-TAP (97.46 kDa) and Wdr83-TAP (57.38 kDa) were grown in YE + 5S and harvested around OD_595_ = 0.8. Extracted proteins were analyzed by SDS-PAGE and Western blotting using anti-tubulin and PAP antibodies. Tubulin (51.15 kDa) was used as a loading control. Full original images of Western blots are shown in Figure S2.

To further support the functional interconnections between the proteins of the Gpl1-Gih35-Wdr83 complex, we asked whether the expression of its components is mutually dependent. We created the strains with single, double or triple deletions of *gpl1*, *gih35* and *wdr83* (Figure S1) and performed the Western blot and RT-qPCR analyses. We detected no significant changes in the mRNA levels of *gpl1*, *gih35* or *wdr83* in the analyzed mutants (Figure 5a). Similarly, we observed the very same levels of expression of Gpl1-TAP, Gih35-TAP and Wdr83-TAP proteins in wild-type cells and in analyzed deletion mutants (Figure 5b). These results also suggest that the loss of Wdr83 from Gih35-TAP purification in the *gpl1*Δ strain (Figure 4) is unlikely due to decreased Wdr83 protein levels.

### 2.6. Deletions of the Gpl1-Gih35-Wdr83 Complex Components Affect the Splicing Efficiency

Gpl1 protein has been previously implicated in the splicing of pre-mRNA. It was found that the deletion of *gpl1* results in a canonical splicing defect with broad increases in pre-mRNA species and decreases in mature mRNA species [44]. However, there was no direct evidence that other components of the Gpl1-Gih35-Wdr83 complex identified here are also involved in pre-mRNA splicing.

**Figure 6:**
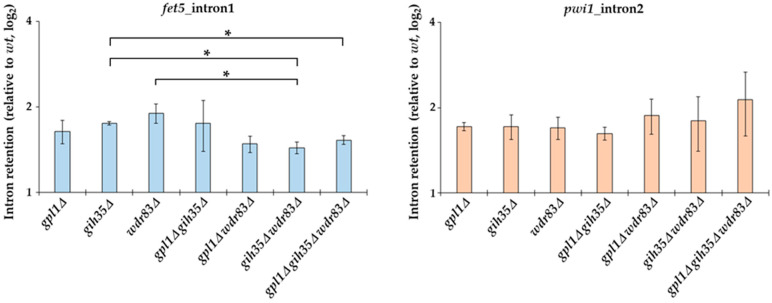
RT-qPCR analysis of splicing efficiency in Gpl1-Gih35-Wdr83 complex deletion mutants. The intron retention relative to wild type (wt level was normalized to 1) is shown for the first intron of fet5 and the second intron of pwi1. The data represent mean values ± SEM of intron retention relative to wild type from three independent biological replicates. Statistical significance of intron retention of studied genes was determined using two-tailed Student’s *t*-test (*p*-values: *—*p* ≤ 0.05).

To find out if Gih35 and Wdr83 affect the splicing processes, we assessed the splicing efficiency of *fet5*_intron1 and *pwi1*_intron2 in Gpl1-Gih35-Wdr83 complex deletion mutants following the retention of the introns relative to wild type. We found that similar to the *gpl1*Δ mutant, both *gih35*Δ and *wdr83*Δ mutants have defects in the splicing of *fet5* and *pwi1*. Deletion of any of the Gpl1-Gih35-Wdr83 complex components resulted in increased intron retention that was reflected by an increase in both *fet5*_intron1 and *pwi1*_intron2 pre-mRNA levels (Figure 6). Interestingly, in contrast to single deletions, we observed a statistically significant decrease in *fet5* pre-mRNA levels when comparing the single *gih35*Δ mutant with the double *gih35*Δ *wdr83*Δ and triple *gpl1*Δ *gih35*Δ *wdr83*Δ mutants or the single *wdr83*Δ mutant with the double *gih35*Δ *wdr83*Δ mutant (*p* ≤ 0.05). Contrary to *fet5*, statistically significant differences between single, double and triple mutants of *gpl1*, *gih35* and *wdr83* were not detected for the splicing of the second intron of *pwi1*.

## 3. Discussion

The spliceosome is a large ribonucleoprotein complex that regulates various biological functions, such as RNA splicing, gene expression, genome stability, chromatin remodeling, etc. [50,51,52]. In addition to the core spliceosome components, there are many proteins and factors that act as splicing activators or repressors and interact with the spliceosome only transiently at specific stages of spliceosome assembly or rearrangement to regulate its activity. Despite their importance, the exact functions and interactomes of most of these regulators still remain uncharacterized.

Previously, it has been shown that over 170 proteins associate with the metazoan spliceosome at some point during the splicing process, with individual assembly intermediates (e.g., spliceosomal B and C complexes) containing significantly fewer (∼110) proteins [53,54]. Similarly, the yeast spliceosomal C complex has been shown to contain ∼50 proteins (compared to ∼110 in metazoan spliceosomal C complexes), while the remaining ∼40 proteins were identified to interact transiently with yeast spliceosome [55]. These findings suggest a highly dynamic nature of spliceosome assembly, rearrangements and regulation, and emphasize the importance of further studies that might help to identify and characterize novel proteins and factors critical for the regulation of fidelity and efficacy of pre-mRNA splicing.

Recently, by searching for novel spliceosome-associated factors in the fission yeast *S. pombe*, we identified Nrl1 protein as a factor that is required not only for proper pre-mRNA splicing of a subset of genes and non-coding RNAs, but also for the maintenance of genome stability by both suppressing R-loops and promoting HR repair. Interestingly, among its top interactors, we have identified the G-patch domain-containing protein Gpl1 [41,43]. It has also been shown that the *gpl1*Δ mutant has canonical splicing defects with broad increases in pre-mRNA species and decreases in mature mRNA species, which suggested that Gpl1 might be a novel factor implicated in the regulation of pre-mRNA splicing [44]. Analyzing the interactome of Gpl1, we found that Gpl1 associates with the splicing factors, thus further supporting its involvement in pre-mRNA splicing [42].

Importantly, several studies have demonstrated that proteins containing the G-patch domain are critical factors regulating the activities of RNA helicases. The ability of G-patch proteins to bind to and modulate the activity of RNA helicases was first observed for the Ntr1-Prp43 complex [34] and subsequently also for several other G-patch proteins and their partner RNA helicases [28,37,56,57,58].

In this study, we decided to characterize in detail the functional interactome of G-patch protein Gpl1. Using the tandem affinity purification strategy, we performed reciprocal isolations of native complexes of Gpl1 and its main interactors represented by putative ATP-dependent RNA helicase Gih35 and WD repeat protein Wdr83. Mass spectrometry analysis of isolated complexes revealed that these three proteins co-purify with each other (Figure 1). To verify this finding, we attempted to analyze the protein–protein interactions between Gpl1, Gih35 and Wdr83 using the Y2H assay. Additionally, as these three proteins are non-essential for cell viability, we created corresponding deletion mutants and purified the complexes of these three proteins to study their protein–protein interactions in vivo, as well as their ability to bind to the spliceosome. These approaches revealed that Gpl1 interacts with both Gih35 and Wdr83 (Figure 2). We also found that Gih35 binds to the middle region (111–259 aa) and Wdr83 to the C-terminal region (259–534 aa) of Gpl1 (Figure 3). Interestingly, we did not observe the specific role of the G-patch domain for the interaction of Gpl1 with Gih35 and Wdr83. This is in contrast to G-patch proteins Ntr1 or ribosome biogenesis factor NKRF, whereby N-terminal or C-terminal domains containing G-patch motifs were shown to be required for interaction with and activation of helicase Prp43/DHX15 [34,59]. On the other side, it has been observed that G-patch domain-containing protein Pfa1 has two distinct binding sites that mediate binding to Prp43 helicase, and only one of them, the N-terminal, contains the G-patch domain [28]. Our finding thus suggests that the G-patch domain of Gpl1 located within its N-terminal part is probably not required for the interaction between Gpl1 and Gih35 helicase. However, it is possible that this domain is important for the ability of Gpl1 to bind to the spliceosome by interacting with other RNA helicases present in the spliceosome or other splicing factors. This is supported by our observation that deletion of *gpl1* resulted in the inability of Gih35 to interact with most of the splicing factors, while deletion of *wdr83* or *gih35* still allows the binding of Gih35 or Wdr83 with some splicing factors, presumably due to the presence of Gpl1 (Figure 4).

Based on these observations, we hypothesized that Gpl1 might function as a bridging and anchoring protein that mediates interactions between Gih35 and Wdr83 and ensures their binding to the spliceosome, thus regulating their spliceosome-related activities. If this was true, and Gpl1, Gih35 and Wdr83 indeed function in splicing of pre-mRNA as part of the Gpl1-Gih35-Wdr83 complex, then deletion of *wdr83* or *gih35* should lead to splicing defects similar to those previously observed in the *gpl1*Δ mutant [44].

Before assessing the splicing defects of *gih35*Δ and *wdr83*Δ mutants, we first checked the effect of the deletion of components of the Gpl1-Gih35-Wdr83 complex on the expression of the remaining components of this complex to exclude the possibility of their mutually dependent regulation. It is known that *gpl1* and *gih35* are located on chromosome I very close to each other (genomic location of *gpl1* is 2122208-2120604 and *gih35* is 2126067-2128359; the distance between these two genes is only 3859 nt). RT-qPCR analysis of gene expression and Western blot analysis of protein levels revealed that neither gene expressions nor protein levels of the Gpl1-Gih35-Wdr83 complex components are affected when particular components of this complex are absent (Figure 5). This finding revealed that proteins forming the Gpl1-Gih35-Wdr83 complex do not affect the expression of each other.

Next, we tested the splicing efficiency of *fet5*_intron1 and *pwi1*_intron2 in Gpl1-Gih35-Wdr83 complex deletion mutants. The *fet5* transcript contains a single 45 nt intron with canonical 5′ splice site (GUAAGU) and branch point (UGCUAAU) sequences. On the other hand, the second intron in *pwi1* is 59 nt long, and has a typical branch point sequence (CAUUAAU) but an atypical 5ʹ splice site sequence (GUACAA), which significantly deviates from the canonical sequence. We found that similarly to the *gpl1*Δ mutant, both *gih35*Δ and *wdr83*Δ mutants have defects in the splicing of *fet5* and *pwi1*. We also found a statistically significant decrease in *fet5* pre-mRNA levels when comparing the single *gih35*Δ mutant with the double *gih35*Δ *wdr83*Δ and triple *gpl1*Δ *gih35*Δ *wdr83*Δ mutants or the single *wdr83*Δ mutant with the double *gih35*Δ *wdr83*Δ mutant. Concerning the splicing of *pwi1*, statistically significant differences between single, double and triple mutants of *gpl1*, *gih35* and *wdr83* were not detected (Figure 6). As such, we can speculate that Gpl1 and Wdr83, in addition to their splicing-related functions within the Gpl1-Gih35-Wdr83 complex, might also be involved in other pathways that affect the level of unspliced pre-mRNA. It is worth mentioning that the Wdr83 interactome, except for the splicing factors, was significantly enriched for all eight proteins forming the chaperonin-containing T-complex (Cct1-Cct8) (Figure 4; Table S2). The CCT complex (chaperonin-containing TCP-1 complex) is known as a large multi-subunit complex that mediates protein folding [60]. This might suggest that the binding of the CCT complex to Wdr83 might be required to promote its proper folding and function, similarly as observed for another WD repeat protein WDR68 [61]. However, we cannot exclude the possibility that CCT proteins are not true Wdr83 interactors and their presence is caused by TAP-tagging of Wdr83.

Taken together, our data suggest that G-patch protein Gpl1, RNA helicase Gih35 and WD repeat domain protein Wdr83 function together as part of a complex, which is important to prevent splicing defects. However, the finding that the G-path portion of Gpl1 was expendable for the interaction of Gpl1 with RNA helicase Gih35 leaves questions on the biological function of this domain. Is the G-patch domain of Gpl1 required for the recruitment of the Gpl1-Gih35-Wdr83 complex to the spliceosome at the right time? Is it important for the regulation of the activity of the Gih35 helicase? Or, is this domain regulating the activity of other spliceosomal helicases? To answer these questions, further research to find out if the Gpl1-Gih35-Wdr83 complex regulates the splicing processes through the RNA helicase Gih35, or if this complex or the G-patch domain of Gpl1 affects the splicing processes by modulating activities of other splicing factors and helicases is needed.

## 4. Materials and Methods

### 4.1. Strains, Media and Growth Conditions

The genotypes of strains, plasmids and sequences of primers used in this study are listed in Table S3. *S. pombe* strains carrying gene deletions or expressing TAP-tagged proteins were constructed as described previously [62,63]. Cells were grown in complete yeast extract medium (YE + 5S; 5.0 g/L yeast extract, 3.0% glucose, 0.1 g/L L-leucine, 0.1 g/L L-lysine hydrochloride, 0.1 g/L L-histidine, 0.1 g/L uracil and 0.15 g/L adenine sulphate). Deletion of genes was confirmed by colony PCR (Figure S1) and RT-qPCR.

### 4.2. Tandem Affinity Purification

Cells expressing TAP-tagged proteins were grown to mid-log phase (OD_595_ = 0.7–0.8) at 32 °C and collected by centrifugation (4000× *g*, 10 min, 4 °C; Z 36 HK, HERLME LaborTechnik, Wehingen, Germany). Yeast cell powders (40 g) were prepared from frozen cell pellets using SPEX SamplePrep 6770 Freezer/Mill (SPEX SamplePrep, Metuchen, NJ, USA) cooled by liquid nitrogen. Proteins were extracted using IPP150 buffer (50 mM Tris pH 8.0, 150 mM NaCl, 10% glycerol, 0.1% NP-40, 1 mM PMSF and complete protease and phosphatase inhibitors) in a ratio of 1 g of yeast cell powder to 3 mL of IPP150 buffer and affinity purified as described previously [43,64]. Briefly, 500 µL of IgG Sepharose™ 6 Fast Flow beads (GE Healthcare, Uppsala, Sweden) was equilibrated with IPP150 buffer, mixed with protein extract and incubated on rotatory wheel for 2 h at 4 °C. Beads with bound proteins were washed with 20 bead volumes of IPP150 buffer followed by washing with 5 bead volumes of TEV cleavage buffer (TCB, 10 mM Tris pH 8.0, 150 mM NaCl, 10% glycerol, 0.1% NP-40, 0.5 mM EDTA and 1 mM DTT). Cleavage step was performed in 2 mL of TCB supplemented with 400 U of Turbo TEV protease (MoBiTec GmbH, Goettingen, Germany) for 2 h at 16 °C. Then, 2 mL of eluate was supplemented with 6 µL of 1 M CaCl_2_ and combined with 6 mL of Calmodulin binding buffer 1 (CBB1, 10 mM Tris pH 8.0, 150 mM NaCl, 10% glycerol, 0.1% NP-40, 1 mM imidazole, 1 mM Mg-acetate, 2 mM CaCl_2_ and 10 mM β-mercaptoethanol). After which, 150 µL of Calmodulin Sepharose™ 4B beads (GE Healthcare, Uppsala, Sweden) was equilibrated with CBB1, combined with mixture of eluate and CBB1 and incubated on rotatory wheel for 2 h at 4 °C. The beads with bound proteins were washed with 10 bead volumes of CBB1 and 5 bead volumes of Calmodulin binding buffer 2 (CBB2, 10 mM Tris pH 8.0, 150 mM NaCl, 1 mM Mg-acetate, 2 mM CaCl_2_ and 1 mM β-mercaptoethanol). The proteins were step-eluted using bead volume of elution buffer (EB, 10 mM Tris pH 8.0, 150 mM NaCl, 1 mM Mg-acetate, 2 mM EGTA and 1 mM β-mercaptoethanol). Eluted fractions were separated by SDS-PAGE and stained using silver staining to follow the elution profile [65]. Eluates from peak fractions were combined and subjected for LC-MS/MS analysis.

### 4.3. LC-MS/MS Analysis

The reduction step was performed by incubating the sample using 5 mM DTT at 60 °C for 30 min. Subsequently, the sample was alkylated by addition of 15 mM iodoacetamide (20 min, RT/in dark). The alkylation reaction was quenched by additional 5 mM DTT. Then, 0.5 µg of modified sequencing grade trypsin (Promega, Madison, WI, USA) was added to the sample and incubated overnight at 37 °C. To stop the trypsin reaction, the mixture was acidified by addition of 0.5% TFA. The peptides were purified by microtip C18 SPE and dried in the Concentrator plus (Eppendorf, Hamburg, Germany). For peptide separation by HPLC, Dionex Ultimate 3000 RSLCnano system (Thermo Scientific, Waltham, MA, USA) was used. After which, 5 μL of sample was loaded onto a trap column (PepMap100 C18, 300 μm × 5 mm, 5-μm particle size) (Thermo Scientific, Waltham, MA, USA) coupled to an EASY-Spray C18 analytical column having integrated nanospray emitter (75 μm × 500 mm, 2 μm particle size) (Thermo Scientific, Waltham, MA, USA). The peptides were separated in 1 h gradient from 3% to 43% B with two mobile phases used: 0.1% FA (*v*/*v*) (A) and 80% ACN (*v*/*v*) with 0.1% FA (B). Spectral datasets were collected by Orbitrap Elite mass spectrometer (Thermo Scientific, Waltham, MA, USA) operating in the data-dependent mode using Top15 strategy for the selection of precursor ions for the HCD fragmentation [66]. Each of the samples was analyzed in at least two technical replicates. Obtained datasets were processed by MaxQuant (version 1.6.17.0) [67] with built-in Andromeda search engine using carbamidomethylation (C) as permanent modification and phosphorylation (STY), acetylation (protein N-terminus) and oxidation (M) as variable modifications. The estimated relative abundance of purified protein was quantified in terms of SAF (spectral abundance factor), which was obtained by dividing spectral counts for a protein by its molecular weight as described previously [68]. Additionally, relative quantities of individual proteins were determined by built-in label-free quantification (LFQ) algorithm MaxLFQ, which provides normalized LFQ intensities for identified proteins [69]. The search was performed against the *S. pombe* protein databases (UniProt, downloaded 12.11.2021 and PomBase, downloaded 17.7.2020).

### 4.4. Yeast-Two-Hybrid (Y2H) Assay

Y2H constructs were prepared using plasmids supplied in the Matchmaker GAL4 2-hybrid system (Clontech, CA, USA). Constructs expressing the protein of interest fused with Y2H DNA-binding domain (BD) were created in the *pAS2-1* vector containing the *TRP1* gene for selection on synthetic dropout tryptophan-deficient media and constructs expressing the protein of interest fused with Y2H activation domain (AD) were made in the *pGADT7* vector containing the *LEU2* gene for selection on synthetic dropout leucine-deficient media. *S. cerevisiae* strain PJ69-4A was co-transformed simultaneously with both BD and AD constructs by the lithium acetate method as described previously [70]. After transformation, cells were plated on synthetic dropout media composed of nitrogen base (1.7 g/L), (NH_4_)_2_SO_4_ (5 g/L), glucose (2%) and a dropout supplements without leucine and tryptophan (SD-L,W) and incubated at 30 °C for 48 h. Colonies growing on SD-L,W were transferred onto synthetic dropout media composed of nitrogen base (1.7 g/L), (NH_4_)_2_SO_4_ (5 g/L), glucose (2%) and a dropout supplements without leucine, tryptophan and adenine (SD-L,W,A) and SD-L,W media supplemented with 80 mg/L 5-bromo-4-chloro-3-indolyl-α-D-galactopyranoside (X-gal, Roche, Basel, Switzerland) and incubated at 30 °C for 48 h.

### 4.5. Analysis of Gene Expression and Splicing Efficacy by RT-qPCR

Cells were inoculated into 50 mL of fresh media (OD_595_ = 0.2) and cultivated at 30 °C to the exponential phase (OD_595_ = 0.5–0.6). Cells were harvested, washed with water and cell pellets were stored at −80 °C. Following, pellets were resuspended in 1xTE, broken down by vortexing with glass beads (3 × 2 min interval) and total RNA was isolated using Thermo Fisher Scientific kits (GeneJET RNA Purification Kit; RapidOut DNA Removal Kit). cDNA was prepared from 1 μg total RNA using Lunascript RT SuperMix Kit (New England BioLabs, Ipswich, MA, USA) according to the manufacturer‘s instructions. For RT-qPCR, FastStart DNA Master SYBR Green master mix (Roche, Carlsbad, CA, USA) was used as instructed.

The transcript levels relative to the wild type were normalized to actin. The ratio of splice isoforms was calculated using formula = 2^(−∆∆Cq), where ∆∆Cq = ∆Cq(intron, mutant)—∆Cq(intron, wild type). Due to the fact that exon Cq values should not be changed in both, mutant and wild-type samples, exon Cq values were used as a reference gene/variant for each sample. Changes in intron splicing of studied mutants were calculated as intron retention values relative to wild type, which were normalized to 1. Statistical significance was determined using two-tailed Student´s *t*-test (*p*-values: *—*p* ≤ 0.05).

### 4.6. Western Blotting

Proteins were separated using 8% SDS-PAGE and transferred to a PVDF membrane (0.45 μm, GE Healthcare, Houston, TX, USA). The membrane was blocked with 5% (*w*/*v*) milk PBS-T (phosphate buffered saline buffer with 0.1% (*v*/*v*) Tween-20) and probed with primary antibodies. The TAP epitope was detected using PAP antibody (rabbit antiperoxidase antibody linked to peroxidase) (Dako, Agilent Technologies, Santa Clara, CA, USA) at 1:20,000 dilution in 5% (*w*/*v*) milk PBS-T. Tubulin was detected using monoclonal anti-α-tubulin primary antibody produced in mouse (Sigma Aldrich, Merck, Darmstadt, Germany) at 1:10,000 dilution in 5% (*w*/*v*) milk PBS-T and rabbit anti-mouse HRP secondary antibody (Sigma Aldrich, Merck, Darmstadt, Germany) at 1:5000 dilution in PBS-T. Pierce ECL Plus Western Blotting Substrate (Thermo Fisher Scientific, Waltham, MA, USA) and Amersham Hyperfilm^TM^ ECL (GE Healthcare, Buckinghamshire, UK) were used for detection.

### 4.7. Predicting Gpl1 Structure Using AlphaFold

The three-dimensional structure of Gpl1 was generated by AlphaFold [48,49] and retrieved from the AlphaFold Protein Structure Database (https://alphafold.com/entry/Q9HE07, accessed on 28 July 2022).

## Data Availability

The data are contained within the article and Supplementary Materials.

## Supplementary Materials

The following supporting information can be downloaded at: https://www.mdpi.com/article/10.3390/ijms232112800/s1.
