# Supplementary material for: Defining the Functional Interactome of Spliceosome-Associated G-Patch Protein Gpl1 in the Fission Yeast Schizosaccharomyces pombe
Source: Int J Mol Sci. 2022 Oct 24;23(21):12800. doi: 10.3390/ijms232112800 (PMC9658070; doi:10.3390/ijms232112800)
Supplement: Supplementary file 1 [file ijms-23-12800-s001.zip › Supplementary data/Figure S1.pptx]

## Slide 1
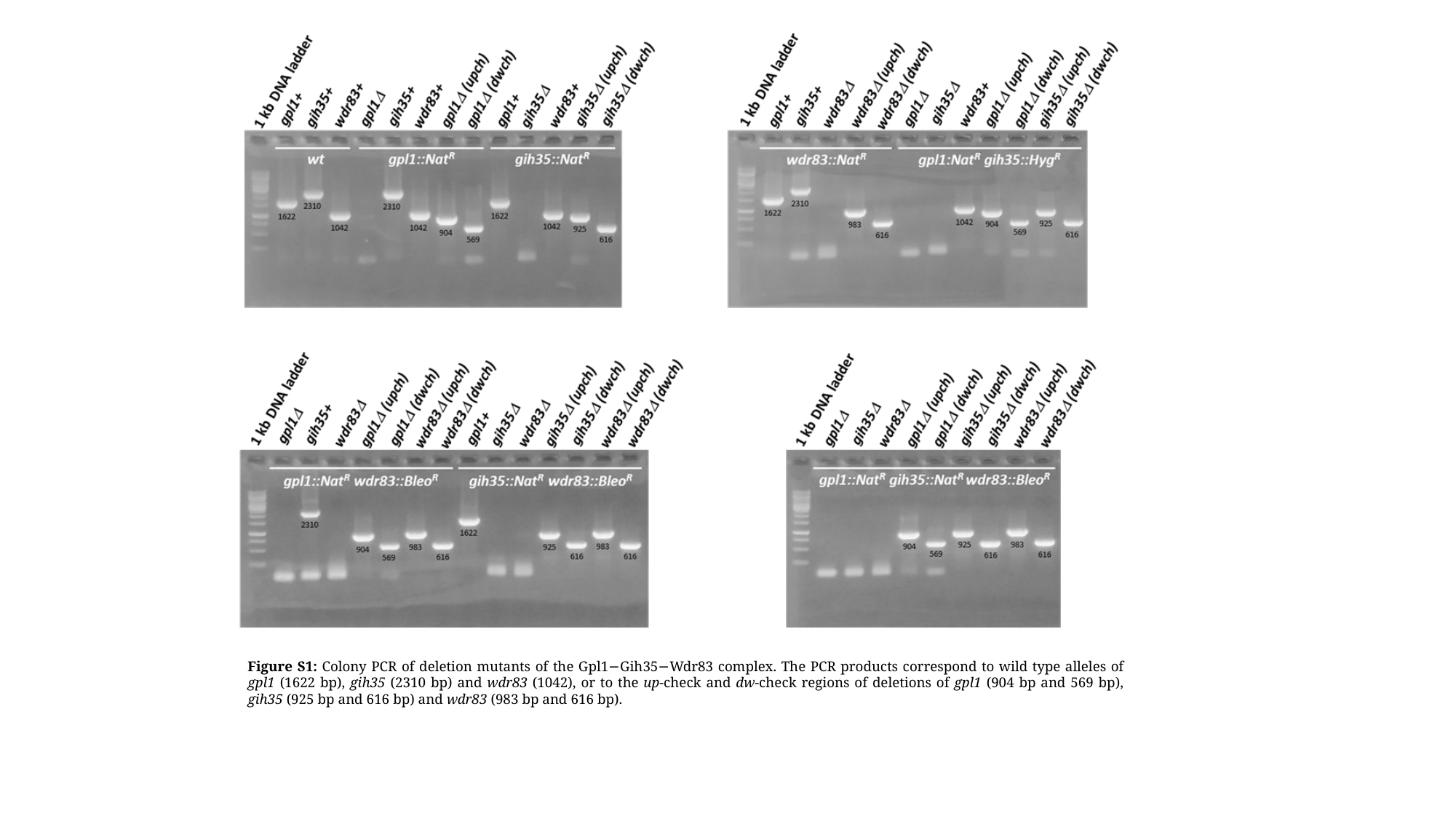

Figure S1: Colony PCR of deletion mutants of the Gpl1−Gih35−Wdr83 complex. The PCR products correspond to wild type alleles of gpl1 (1622 bp), gih35 (2310 bp) and wdr83 (1042), or to the up-check and dw-check regions of deletions of gpl1 (904 bp and 569 bp), gih35 (925 bp and 616 bp) and wdr83 (983 bp and 616 bp).
