# Supplementary material for: Defining the Functional Interactome of Spliceosome-Associated G-Patch Protein Gpl1 in the Fission Yeast Schizosaccharomyces pombe
Source: Int J Mol Sci. 2022 Oct 24;23(21):12800. doi: 10.3390/ijms232112800 (PMC9658070; doi:10.3390/ijms232112800)
Supplement: Supplementary file 1 [file ijms-23-12800-s001.zip › Supplementary data/Figure S2.pptx]

## Slide 1
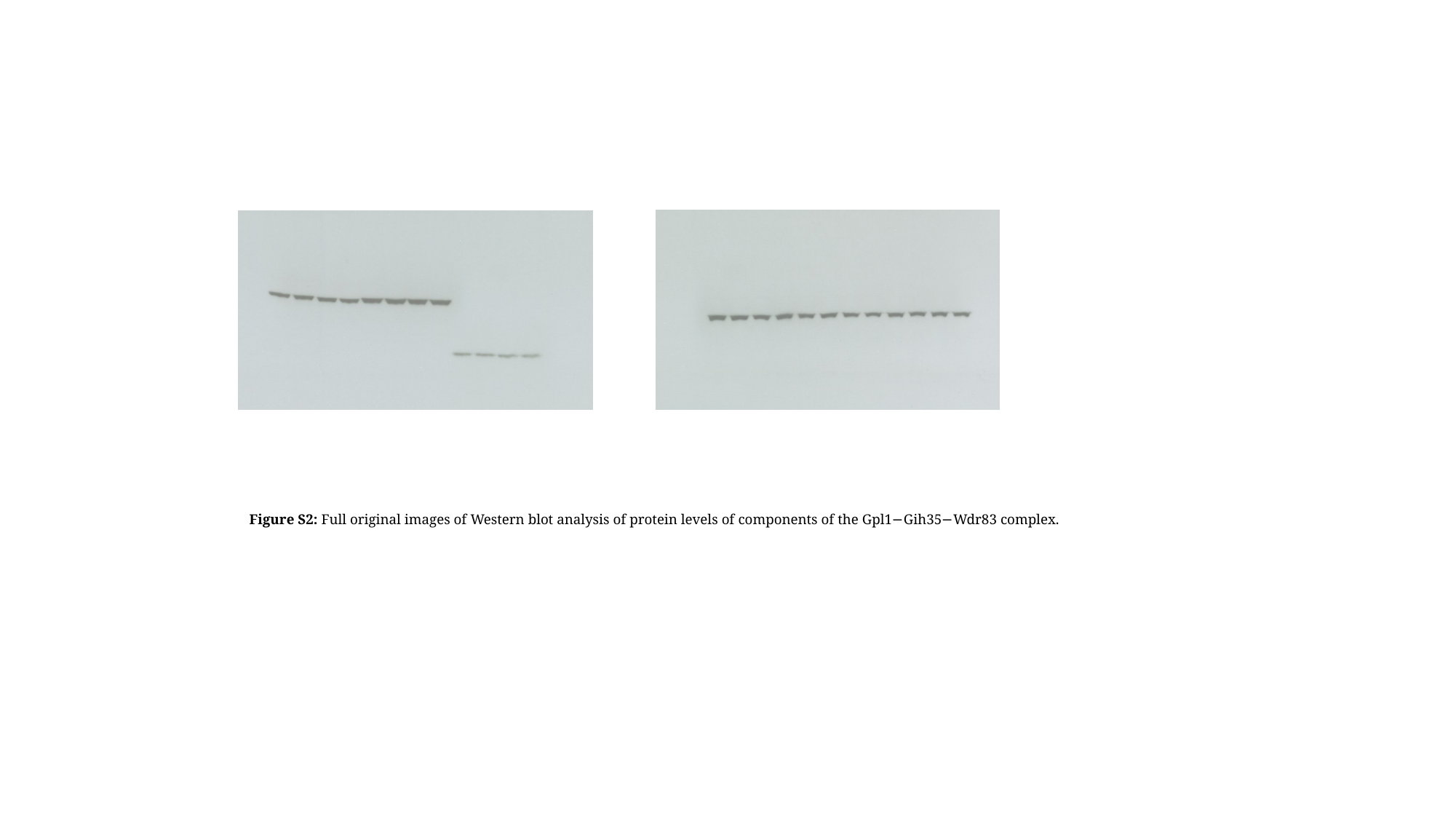

Figure S2: Full original images of Western blot analysis of protein levels of components of the Gpl1−Gih35−Wdr83 complex.
